# Hand hygiene improvement of individual healthcare workers: results of the multicentre PROHIBIT study

**DOI:** 10.1186/s13756-022-01148-1

**Published:** 2022-10-05

**Authors:** Tjallie van der Kooi, Hugo Sax, Hajo Grundmann, Didier Pittet, Sabine de Greeff, Jaap van Dissel, Lauren Clack, Albert W. Wu, Judith Davitt, Sofia Kostourou, Alison Maguinness, Anna Michalik, Viorica Nedelcu, Márta Patyi, Janja Perme Hajdinjak, Milena Prosen, David Tellez, Éva Varga, Fani Veini, Mirosław Ziętkiewicz, Walter Zingg, the PROHIBIT consortium

**Affiliations:** 1 RIVM National Institute for Public Health and the Environment, Bilthoven, The Netherlands; 2 Clinic for Infectious Diseases and Hospital Hygiene, University Hospital Zürich, Rämistrasse 100, 8091 Zurich, Switzerland; 3 Medical Center – University of Freiburg, Freiburg, Germany; 4 University of Geneva Hospitals, Geneva, Switzerland; 5 WHO Collaborating Centre on Infection Prevention and Control and Antimicrobial Resistance, Geneva, Switzerland; 6 Center for Health Services and Outcomes Research, Johns Hopkins University Bloomberg School of Public Health, Baltimore, MD USA; 7 Galway University Hospital, Galway, Ireland; 8 Evangelismos Hospital, Athens, Attica Greece; 9 St. Michaels Hospital, Dún Laoghaire, Ireland; 10 Faculty of Health Sciences, University of Bielsko-Biala, Bielsko-Biala, Poland; 11 Emergency Institute for Cardiovascular Diseases “Prof. C.C. Iliescu”, Bucharest, Romania; 12 Bács-Kiskun Megyei Kórház (County Teaching Hospital), Kecskemet, Hungary; 13 University Medical Centre Ljubljana, Ljubljana, Slovenia; 14 Hospital Vall d’Hebron, Barcelona, Catalunya Spain; 15 John Paul II Hospital, Kraków, Poland; 16 Medical College Jagiellonian University, Kraków, Poland

**Keywords:** Hand hygiene, Activity index, Individual, Intervention, Intensive care, Multicentre

## Abstract

**Background:**

Traditionally, hand hygiene (HH) interventions do not identify the observed healthcare workers (HWCs) and therefore, reflect HH compliance only at population level. Intensive care units (ICUs) in seven European hospitals participating in the “Prevention of Hospital Infections by Intervention and Training” (PROHIBIT) study provided individual HH compliance levels. We analysed these to understand the determinants and dynamics of individual change in relation to the overall intervention effect.

**Methods:**

We included HCWs who contributed at least two observation sessions before and after intervention. Improving, non-changing, and worsening HCWs were defined with a threshold of 20% compliance change. We used multivariable linear regression and spearman’s rank correlation to estimate determinants for the individual response to the intervention and correlation to overall change. Swarm graphs visualized ICU-specific patterns.

**Results:**

In total 280 HCWs contributed 17,748 HH opportunities during 2677 observation sessions. Overall, pooled HH compliance increased from 43.1 to 58.7%. The proportion of improving HCWs ranged from 33 to 95% among ICUs. The median HH increase per improving HCW ranged from 16 to 34 percentage points. ICU wide improvement correlated significantly with both the proportion of improving HCWs (ρ = 0.82 [95% CI 0.18–0.97], and their median HH increase (ρ = 0.79 [0.08–0.97]). Multilevel regression demonstrated that individual improvement was significantly associated with nurse profession, lower activity index, higher nurse-to-patient ratio, and lower baseline compliance.

**Conclusions:**

Both the proportion of improving HCWs and their median individual improvement differed substantially among ICUs but correlated with the ICUs’ overall HH improvement. With comparable overall means the range in individual HH varied considerably between some hospitals, implying different transmission risks. Greater insight into improvement dynamics might help to design more effective HH interventions in the future.

**Supplementary Information:**

The online version contains supplementary material available at 10.1186/s13756-022-01148-1.

## Introduction

Healthcare-associated infections (HAIs) affect on average 6% of hospitalized patients in Europe [1, 2]. Patients in hospitals are at increased risk of acquiring HAIs mainly because of invasive procedures. The proximity to other patients and frequent healthcare contacts facilitate the transmission of pathogens, and the use of broad-spectrum antibiotics increases the burden of multidrug-resistant microorganisms (MDROs) in hospital settings [3]. Hand hygiene (HH) is the most basic and essential element in the prevention of cross-transmission. Although widely promoted, HH compliance in intensive care units (ICU) remains on average 40–50% [4–7], and to sustain achieved improvements remains challenging [8–11].

All but a few small studies [12–17] on HH compliance report only pooled data across all healthcare workers (HCW). Thus there is little data on the contribution of individuals to the overall response to behavioural interventions. Individual HH compliance is relevant because average compliance does not reflect variations among HCWs and thus, might not capture overall transmission risk. Moreover, data on changes in individual HH compliance could provide insights into barriers and facilitators of a HH intervention. In the Prevention of Hospital Infections by Intervention and Training (PROHIBIT) intervention study, HH compliance was observed at the individual HCW level during baseline and intervention study periods. We analysed these data to better understand the determinants and dynamics of individual change in HH compliance in relation to the overall intervention effect.

## Methods

### Methods of the PROHIBIT intervention study

The PROHIBIT intervention study tested two interventions to prevent central venous catheter bloodstream infection (CRBSI) in Intensive Care Units (ICUs) in European acute care hospitals: a central venous catheter (CVC) insertion strategy, and a HH improvement strategy. Several other work packages addressed the topic of HAI prevention more widely [18–22]. After a baseline period of at least six months, three ICUs of 14 hospitals in 11 European countries were randomly allocated every three months to start with one of the two intervention strategies or both [7]. The method and results of this step wedge randomised controlled intervention study have been reported in detail elsewhere [7, 23].

In brief, each ICU appointed one dedicated on-site investigator and one study nurse to the project. PROHIBIT offered reimbursement of a 0.5 full-time equivalent study nurse. Three to six months before the start of the intervention, local study nurses and/or infection control physicians, anaesthetists, and intensivists, depending on the intervention, attended a two-day PROHIBIT workshop on best practices and implementation science. For the HH improvement strategy, PROHIBIT used the WHO HH training and campaign materials (http://www.who.int/gpsc/5may/tools/en/). Hand hygiene promotion included educational sessions and bedside training. In addition, the ICUs displayed posters and/or other reminders in the workplace and participants came up with various additional promotion activities. Hand hygiene compliance was measured by direct observation according to the WHO observation method [24]. PROHIBIT study nurses, most often infection prevention and control (IPC) or ICU nurses with IPC responsibilities, were trained in the methodology of direct HH observation at the University of Geneva Hospitals, Switzerland. Hand hygiene observations were randomized for date (weekdays), time slot (08–12:00, 12:00–16:00 and 16:00–20:00), and ICU bed [7]. One observation session could include observations of multiple HCWs. However, to avoid missing HH opportunities, observers were not allowed to observe more than three HCWs in one session. Hand hygiene opportunities were stratified by observation sessions and by the five WHO indications for HH [24]. HH compliance was calculated as the proportion of HH opportunities met by a HH action. Individual HCWs identity was recorded using a four-letter code, based on their given and family name, where needed retrieved from the badge and, if that proved impossible, by asking the HCW.

During the intervention on site investigators received quarterly feedback reports on the average HH compliance in their ICU and individual HCWs on their HH compliance after being observed, but not during baseline [7]. HCW codes were used for statistical analysis only.

### Study population of the present analysis

Ten of the 14 ICUs agreed to capture the HCWs’ identity during HH observations. Seven of these ICUs implemented the HH intervention, either alone or in combination with the CRBSI-prevention strategy and were included in the present analysis as *study ICUs*. Only data of HCWs with at least two observation sessions, during both baseline and intervention periods, were included for the individual analysis (‘*study HCWs*’).

### Definitions

HCWs were grouped according to the change in their HH compliance between baseline and intervention.

‘*Improving HCW’* were defined by having improved compliance by at least 20%, ‘*Worsening HCW’* decreased by at least 20%, and ‘*Non-changing HCW’* changed less than 20%, if at all. The 20%-threshold was chosen retrospectively based on the rounded pooled mean change among all HCWs. An ‚*activity index* ‘, defined by number of hand hygiene opportunities per hour of observation, was defined as a proxy for the intensity of care [25].

### Analysis plan and statistics

To meet our study scope, we chose four analytical models.

In Model 1, we evaluated the extent to which changes in the individual HH compliance between study periods were associated with ICU characteristics. We calculated Spearman rank correlation coefficients of the proportion of *Improving HCWs* with a) the nurse-to-patient ratio and b) the pooled mean baseline HH compliance of all HCWs.

In Model 2, we assessed a potential association between the intervention effect on the individual HCW and the overall ICU. We calculated the Spearman’ rank correlation coefficients for the proportion and median improvement of *Improving HCWs* with the pooled change in HH compliance of all HCWs.

In Model 3, we tested the potential association of the change in HH compliance for each individual HCW (measured as change in percentage points (pp); outcome variable) with HCW characteristics (i.e., professional category, baseline compliance) and contextual factors (i.e., activity index, ICU type, ICU nurse- to-patient ratio, and proportion of improving HCWs). We used a generalized linear mixed model (GLMM) with a normal distribution, allowing for clustering at the ICU level. Variables with a *P *value < 0.25 in the univariable analysis were included in the multivariable model using manual backward selection. The proportion of explained variation (R^2^) was calculated for this model.

All changes in HH compliance were calculated as relative proportions (%) or differences in percentage points (pp), using mid-P exact tests to test for significance. We used SAS 9.4 (SAS Institute Inc., Cary, United States) for all statistical analyses.

In Model 4, we created a swarm plot with individual HH compliance at baseline and intervention for each HCW, in each category (improving, non-improving, worsening HCWs), and a bar diagram of the range in HH between HH sessions for each HCW, during the baseline and intervention phase, for visual display of individual HCW compliance patterns in the seven study ICUs.

## Results

### Study intensive care units and healthcare worker population

Three of the seven study hospitals were university affiliated, two had > 50,000 admissions per year, two between 30,000 and 50,000, and three < 30,000 admissions per year. The median number of ICU beds per hospital was 17 (range 10–40). The median nurse-to-patient ratio in the ICU during day shifts was 0.5 (range 0.29–1.00; Additional file 1: Table S1) at baseline, i.e., one nurse for two patients. The median activity index in the ICUs was 9.0 (IQR, 6.0–15.0) HH opportunities per hour. According to the inclusion criteria, 280 study HCWs (58% nurses, 20% doctors, 18% auxiliary nurses, 4% other HCWs) contributed 17,748 HH opportunities during 2677 observation sessions with a median number of sessions, respectively opportunities, per HCW of 4 [interquartile range (IQR), 2–6], respectively 15 (IQR 10–23), during baseline and 10 (IQR 5–15), respectively 39 (IQR 18–58.5), during intervention. During baseline 365 HCW and during intervention another 623 HCWs were excluded because they did not meet the inclusion criteria of at least two observation sessions per study period.

### Hand hygiene compliance

**Fig. 1:**
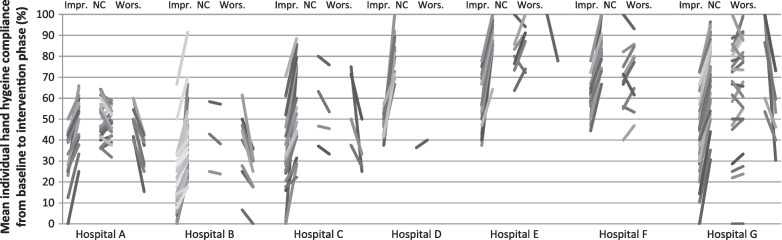
Individual change of hand hygiene compliance, stratified by intensive care unit. Each line represents one HCW. Impr., improving healthcare workers; NC, Non-changing healthcare workers; Wors., Worsening healthcare workers

The pooled mean HH compliance of study HCWs increased significantly from 43.1% during baseline to 58.7% during intervention (Table 1). Similarly, the compliance of the 365 excluded HCWs was 43.1% during baseline. Overall HH compliance of study HCWs and non-study HCWs increased from 43.1% to 60.8% (Additional file 1: Table S2, 61.0% for the excluded 623 HCWs). For *Improving HCWs*, *Worsening HCWs*, and *Non-changing HCWs* HH compliance changed from 35 to 57%, 50% to 41%, and 59% to 66%, respectively. Individual HH compliance per HCW for the entire study population is shown in Fig. 1 and for each study ICU in Additional file 1: Figs. S1 and S2.

**Table 1:** Hand hygiene compliance per hospital during baseline and intervention

Hospital	Number of HCWs	HH compliance (%) during baseline	HH compliance (%) after intervention	Percentage points change in overall HH compliance (95% confidence interval)	Proportion of improving HCWs (%)	Proportion of non-changing HCWs (%)	Proportion of worsening HCWs (%)
A	52	44.1	48.7	4.7 (0.84–8.5)	32.7	51.9	15.4
B	64	16.7	34.7	18.0 (15.1–20.9)	82.8	4.7	12.5
C	28	36.6	49.0	12.4 (8.3–16.5)	71.4	14.3	14.3
D	21	47.1	78.6	31.5 (24.9–38.2)	95.2	4.8	0.0
E	25	62.7	90.9	28.3 (22.6–33.9)	68.0	28.0	4.0
F	36	62.2	79.8	17.5 (13.4–21.7)	61.1	38.9	0.0
G	54	55.5	69.2	13.7 (10.1–17.3)	46.3	42.6	11.1
Total	280	43.1	58.7	15.6 (14.0–17.2)	62.1	28.2	9.6

*HCW* healthcare worker, *HH* hand hygiene

### Model 1: The proportion of Improving Healthcare workers and associated factors per ICU

The overall proportion of *Improving HCWs* was 62.1% with an inter-ICU range of 32.7% to 95.2% (Table 1). Per ICU, the proportion of *Improving HCWs* was not significantly associated with the nurse-to-patient ratio (ρ 0.23; CI − 0.64 to 0.84) or the overall baseline compliance (ρ 0.37; − 0.86 to 0.54).

The average improvement in HH compliance was negatively associated with the activity index (Additional file 1: Table S1).

### Model 2: Association of the intervention effect between individual HCWs and the overall ICU

**Fig. 2:**
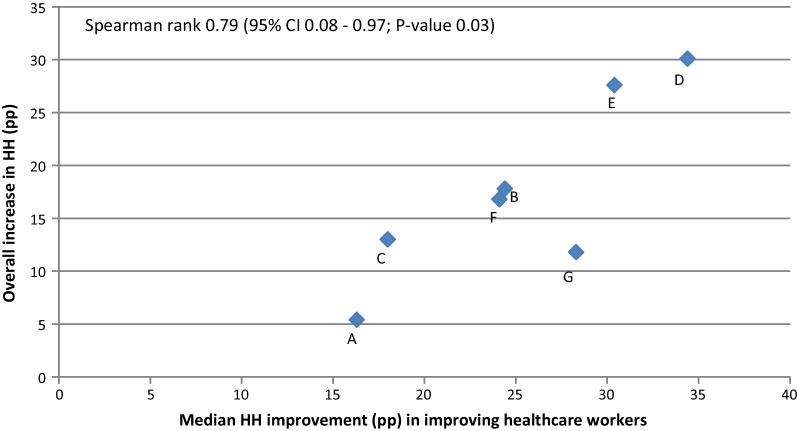
Correlation between median hand hygiene improvement of improving healthcare workers and overall improvement in hand hygiene compliance per hospital. HH, hand hygiene; pp, percentage points

**Fig. 3:**
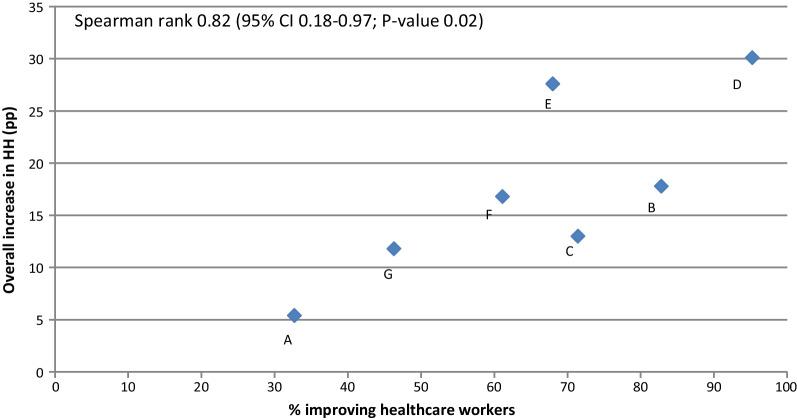
Correlation between the proportions of improving healthcare workers and the overall improvement in hand hygiene compliance per hospital. HH, hand hygiene; pp, percentage points

The median increase in HH compliance of *Improving HCWs* per ICU ranged from 16 to 34 pp (Additional file 1: Fig. S2) and was significantly associated with the overall improvement among all HCWs in the corresponding ICU (ρ 0.79; CI 0.08–0.97) (Fig. 2). The overall proportion of *Improving HCWs* was 62.1% with an inter-ICU range of 32.7% to 95.2% (Table 1). This proportion of *Improving HCWs* per ICU was associated with the overall HH improvement among all HCWs [Spearman rank correlation (ρ) 0.82; 95% confidence interval (CI), 0.18–0.97] (Table 1, Fig. 3).

### Model 3: Factors associated with individual HH compliance change between study periods

In multivariable analysis, a higher nurse-to-patient ratio was positively associated, and professional category ‘medical doctor/ students’, higher activity index, and higher individual baseline HH compliance were negatively associated with individual changes in HH compliance between study periods (Table 2). The multivariable regression model explained 43% of the variance in individual changes in HH compliance between study periods (marginal R2), 22% due to the independent variables (fixed effects) and 21% due to nested clustering on the HCWs and hospital level (random effects).

**Table 2:** Univariable and multivariable estimates of the effect on the change of hand hygiene compliance

	Univariable estimate			Multivariable estimate		
	pp	CI 95%	*p* value	pp	CI 95%	*p* value
Professionals						
Nurse/student nurse	Reference			Reference		
Auxiliaries	0.8	− 4.1 to 5.7	0.74	0.4	− 3.5 to 4.4	0.84
Medical doctors/students	− 2.4	− 7.3 to 2.5	0.34	− 5.7	− 9.7 to − 1.7	0.005
Other healthcare professionals	6.3	− 2.6 to 15.2	0.17	4.1	− 3.5 to 11.7	0.29
Type of ICU						
Medical/surgical	Reference					
Cardiosurgery	− 7.8	− 25.7 to 10.1	0.39			
Vascular surgery	− 6.3	− 23.1 to 10.6	0.47			
Activity index^a,b^						
Per one extra opportunity/h	− 0.6	− 0.8 to − 0.4	< 0.0001	− 0.6	− 0.8 to − 0.4	< 0.0001
Baseline compliance^b^						
Per PP higher compliance	− 0.6	− 0.7 to − 0.5	< 0.0001	− 0.6	− 0.7 to − 0.5	< 0.0001
Nurse-to-patient ratio^b^						
Per PP increase	0.2	− 0.1 to 0.5	0.25	0.5	0.07–0.8	0.02

CI 95%, 95% confidence interval; pp, percentage point

^a^Activity index of the sessions during the intervention period

^b^Differences of mean (centered)

### Model 4

Although lower baseline compliance overall was associated with a larger increase in HH compliance, it can seen in Fig. 1 that *Improving HCWs* with low baseline HH compliance remained lower in intervention compared to *Improving HCWs* who were already high during baseline. Figure 1 shows different HCW compliance patterns and changes across the study ICUs. In some centres the variability in HH compliance among individual HCWs increased from baseline to intervention – resulting from higher variability in compliance between HCWs (Additional file 1: Fig. S1) and/or between sessions of individual HCWs (Additional file 1: Fig. S3).

## Discussion

The goal of this study was to understand the determinants and dynamics of individual HH compliance in response to an intervention. The intervention was a success, HH compliance increased significantly and importantly, overall and in each ICU. Individual compliance change was positively correlated with the overall change per ICU. However, we found large inter-ICU and inter-individual differences in the observed HH compliance and their dynamics. In some ICUs the overall result of the intervention was produced by almost exclusively improving HCWs, while in other ICUs the contribution of improving HCWs was to some extent offset by worsening or non-changing HCWs. We also found that individual compliance change was positively associated with a higher nurse-to-patient ratio, and negatively associated with a higher activity index, with being a physician or medical student, and higher baseline HH compliance.

This study is important, because understanding the patterns and dynamics of individual HH compliance in response to an intervention might help to better tailor improvement efforts. When individual responses to the intervention diverge strongly one might suspect factors that concern HCWs individually. In the contrary case, where all HCWs respond with a similar but smaller increase in HH compliance, contributing to the same overall result, systemic factors might be sought and addressed. For example, in addition to a high baseline compliance, these might include a flawed handrub dispenser placement, a high activity index, or a low nurse-to-patient ratio. Indeed, a higher nurse-to-patient ratio was independently associated with a higher HH improvement in this study. Moreover, the ICU with the highest nurse-to-patient ratio (ICU E) was the only one in which the activity index was not negatively associated with HH compliance. This suggests that the nurse-to-patient ratio is a relevant variable to target as a risk for low HH compliance. The negative effect of a high workload on HH compliance has been reported before [25–28]. Scheithauer et al. demonstrated an inverse relationship between the daily workload and HH [29], and Lee et al. found a positive association between nurse-to-patient ratio and HH compliance [16]. Data collected by Hansen et al. demonstrate a nurse to patient ratio of 0.5 during dayshifts to be typical for European ICUs, with national averages ranging from 0.3 to 0.9 [30].

Our study reveals considerable variability in HH compliance between individual HCWs, in some ICUs more than in others. The few previous studies that have employed individualized observation found similar between-HCW variability [12–17, 31]. However, these were either based on a small number of HCWs or did not perform an intervention. Estimating from Fig. 1 and Additional file 1: Fig. S1, this between-HCW variability seems to increase in some centres, suggesting differences in the individual response to the intervention. Personal perceptions, mental models, motivation, and work organisation can influence the individual HH behaviour of each HCW and, in consequence, also their response to promotional exposure [31–34]. Early recognition of these individual factors could help in customizing HH improvement strategies to a range of typical behavioural profiles. Methods from psychology and implementation science may be helpful to tailor improvement strategies to prospectively identified determinants of HH [35–37].

Data on the level of HH compliance needed to prevent cross-transmission are limited to modelling studies. Three reports identified a “threshold” of mean HH compliance above which pathogen transmission and infections would start to decline to be 48%, 66% and 87%, respectively, always assuming that each HH action results in a total eradication of pathogen transmission[38–40]. Models taking into account less than 100% efficacy, conclude that no level of HH can be identified as “good enough” to prevent transmission[41–43]. Most importantly, models have demonstrated that the distribution of HH compliance among HCWs in a population affects the ensuing transmission risk. A single HCW with low HH compliance could play a significant role in pathogen transmission, especially if such a low-performing HCW provides care to many patients consecutively. This effect was demonstrated in two agent-based models by Temime et al. and by Hornbeck et al. [44, 45]. Exemplarily, the proportion of low-performing staff in our study were predominantly doctors who typically deliver care to many different patients. This ‘weakest link’ mechanism challenges the usefulness of pooled means of HH compliance infectious risk in each care unit. Our real-world data support the idea that similar pooled mean HH compliance rates between observed settings can be the result of quite different distributions of high- and low-performing HCWs.

This study has limitations. First, not all observed HCWs could be included in the analysis due to the required number of observations. This could have led to an overrepresentation of permanent staff. Both the pooled baseline and intervention compliance were, however, comparable between the group of study HCWs and the excluded HCWs.

Second, the definition of *Improving HCWs* as those with ≥ 20% HH improvement precluded the inclusion of HCWs with > 80% compliance at baseline in this category. To circumvent this problem, we evaluated the possibility of using the change in *non-*compliance, rather than compliance to distinguish HCWs. This resulted in slightly higher correlations and similar effect estimates in the univariable regression model. We therefore decided to remain with the traditional definition of HH compliance. Third, the chosen cut-off value of 20% to distinguish HCWs into the three HH compliance change categories was somewhat arbitrary. However, a multivariable model with a cut-off value of 10% provided similar results (data not shown). Fourth, some of the observed between-HCW variability could be explained by chance due to a limited number of HH observation sessions per HCW and a limited number of opportunities per session. However, our study is the largest of its kind to date and demonstrates the feasibility and benefit of this approach. It might take an advanced automatic HH monitoring system to collect a larger number of opportunities per identified HCW. Finally, like other HH observation studies, observer and observation biases cannot be entirely excluded, especially a desirability bias by the observers also being involved in the promotion of the intervention, and observation bias, also known as Hawthorne effect [46–48]. However, given the long study duration of 30 months and the focus on improvement dynamics, it is likely that neither biases influenced the results to a degree that would invalidate our findings.

## Conclusions

Both the proportion of improving HCWs and the median of individual HH improvement differed substantially among hospitals. Both measures were associated with the overall success of the intervention. However, the patterns and dynamics of individual HH compliance varied considerably among ICUs, and could potentially result in different risk of pathogen transmission. Being a nurse, a low individual baseline HH compliance, a lower ICU-level activity index, and a favourable nurse-to-patient ratio were associated with a higher individual HH compliance improvement. Data on individual HH compliance could advance our understanding of improvement dynamics and inform better intervention strategies. Collecting individual level HH data should be seriously considered in future HH research, especially in the design of interventions.

## Supplementary Information

**Additional file 1: Table S1.** Association between hand hygiene and activity index during baseline and intervention; **Table S2.** Overall (i.e. for all HCWs) hand hygiene compliance per hospital, during baseline and the intervention period; **Figure S1.** Boxplot (median, quartiles and ranges) of individual HH compliance both during baseline and intervention period; **Figure S2.** Boxplot (median, quartiles and ranges) of hand hygiene increase (in percentage points) in the group of improving HCWs; **Figure S3.** Variability within improving HCWs, measured as the range between the observation session with the lowest compliance and the session with the highest compliance for each HCW.

## Data Availability

The data are stored with the RIVM. Applications to use the data for further research can be sent to Dr. Walter Zingg (e-mail, Walter.Zingg@usz.ch).

## Abbreviations

ABHR: Alcohol-based handrub
CRBSI: Catheter-related bloodstream infection
CVC: Central venous catheter
HAI: Health care-associated infection
HCW: Healthcare worker
HH: Hand hygiene
ICU: Intensive care unit
IPC: Infection prevention and control
IQR: Interquartile range
MDRO: Multidrug-resistant microorganism
pp: Percentage point
PROHIBIT: Prevention of hospital infection by intervention and training
WHO: World Health Organization
