# Correction: Hand hygiene improvement of individual healthcare workers: results of the multicentre PROHIBIT study

**DOI:** 10.1186/s13756-023-01217-z

**Published:** 2023-02-19

**Authors:** Tjallie van der Kooi, Hugo Sax, Hajo Grundmann, Didier Pittet, Sabine de Greeff, Jaap van Dissel, Lauren Clack, Albert W. Wu, Judith Davitt, Sofia Kostourou, Alison Maguinness, Anna Michalik, Viorica Nedelcu, Márta Patyi, Janja Perme Hajdinjak, Milena Prosen, David Tellez, Éva Varga, Fani Veini, Mirosław Ziętkiewicz, Walter Zingg, the PROHIBIT consortium

**Affiliations:** 1 RIVM National Institute for Public Health and the Environment, Bilthoven, The Netherlands; 2 Division of Infectious Diseases and Hospital Epidemiology, University Hospital Zurich, Rämistrasse 100, 8091 Zurich, Switzerland; 3 Medical Center – University of Freiburg, Freiburg, Germany; 4 University of Geneva Hospitals, Geneva, Switzerland; 5 WHO Collaborating Centre on Infection Prevention and Control and Antimicrobial Resistance, Geneva, Switzerland; 6 Center for Health Services and Outcomes Research, Johns Hopkins University Bloomberg School of Public Health, Baltimore, MD USA; 7 Galway University Hospital, Galway, Ireland; 8 Evangelismos Hospital, Athens, Attica Greece; 9 St. Michaels Hospital, Dún Laoghaire, Ireland; 10 Faculty of Health Sciences, University of Bielsko-Biala, Bielsko-Biala, Poland; 11 Emergency Institute for Cardiovascular Diseases “Prof. C.C. Iliescu”, Bucharest, Romania; 12 Bács-Kiskun Megyei Kórház (County Teaching Hospital), Kecskemet, Hungary; 13 University Medical Centre Ljubljana, Ljubljana, Slovenia; 14 Hospital Vall d’Hebron, Barcelona, Catalunya Spain; 15 John Paul II Hospital, Kraków, Poland; 16 Medical College Jagiellonian University, Kraków, Poland; 17 Department of Infectious Diseases, Bern University Hospital and University of Bern, Bern, Switzerland

**Correction: Antimicrobial Resistance & Infection Control (2022) 11:123** **https://doi.org/10.1186/s13756-022-01148-1**

The original article [1] omitted an affiliation for Hugo Sax which has since been reinstated.

The affiliation for Walter Zingg has also had a minor amendment.
