# Supplementary material for: Hand hygiene improvement of individual healthcare workers: results of the multicentre PROHIBIT study
Source: Antimicrob Resist Infect Control. 2022 Oct 5;11:123. doi: 10.1186/s13756-022-01148-1 (PMC9536014; doi:10.1186/s13756-022-01148-1)
Supplement: Supplementary file 1 — Additional file 1: Table S1. Association between hand hygiene and activity index during baseline and intervention; Table S2. Overall (i.e. for all HCWs) hand hygiene compliance per hospital, during baseline and the intervention period; Figure S1. Boxplot (median, quartiles and ranges) of individual HH compliance both during baseline and intervention period; Figure S2. Boxplot (median, quartiles and ranges) of hand hygiene increase (in percentage points) in the group of improving HCWs; Figure S3. Variability within improving HCWs, measured as the range between the observation session with the lowest compliance and the session with the highest compliance for each HCW. [file 13756_2022_1148_MOESM1_ESM.docx]

Hand hygiene improvement of individual healthcare workers – Results of the multicentre PROHIBIT study – Supplementary material

**Supplementary Table 1.** Association between hand hygiene and activity index during baseline and intervention.

|  | **Baseline** | | | | **Intervention** | | | |
| --- | --- | --- | --- | --- | --- | --- | --- | --- |
| Hospital | Nurse to patient ratio | HH  compliance | *PP change per 1 PP increase in activity index (p-value) | | Nurse to patient ratio | HH  compliance | *PP change per 1 PP increase in activity index (p-value) | |
| A | 0.50 | 44.1 | 0.82 | (0.0007) | 0.50 | 48.7 | -0.55 | (0.042) |
| B | 0.33 | 16.7 | 0.20 | (0.60) | 0.25 | 34.7 | -0.95 | (< 0.0001) |
| C | 0.33 | 36.6 | -0.57 | (0.18) | 0.50 | 49.0 | -1.1 | (0.032) |
| D | 0.67 | 47.1 | 0.07 | (0.81) | 0.67 | 78.6 | -1.1 | (0.0003) |
| E | 1.0 | 62.7 | -2.0 | (0.005) | 0.75 | 90.9 | 0.72 | (0.003) |
| F | 0.50 | 62.2 | -2.5 | (< 0.0001) | 0.50 | 79.8 | -1.9 | (0.0001) |
| G | 0.29 | 55.5 | -0.31 | (0.37) | 0.25 | 69.2 | -0.25 | (0.041) |

*Univariable analysis, clustered on healthcare worker level
HH, hand hygiene; PP, percentage point

**Supplementary Table 2**. Overall (i.e. for all HCWs) hand hygiene compliance per hospital, during baseline and the intervention period

| **Hospital** | **Baseline** | | | **Intervention** | | | **Change** | |
| --- | --- | --- | --- | --- | --- | --- | --- | --- |
|  | opportunities | actions | compliance | opportunities | actions | compliance | **PP** | CI95% |
| A | 1284 | 549 | 42.8 | 3325 | 1602 | 48.2 | **5.4** | 2.2 - 8.6 |
| B | 1417 | 240 | 16.9 | 4805 | 1669 | 34.7 | **17.8** | 15.4 - 20.2 |
| C | 1285 | 444 | 34.6 | 1665 | 793 | 47.6 | **13.1** | 9.5 - 16.6 |
| D | 572 | 279 | 48.8 | 3961 | 3125 | 78.9 | **30.1** | 25.8 - 34.4 |
| E | 815 | 505 | 62.0 | 1488 | 1333 | 89.6 | **27.6** | 23.9 - 31.3 |
| F | 810 | 507 | 62.6 | 1591 | 1263 | 79.4 | **16.8** | 12.9 - 20.7 |
| G | 1126 | 625 | 55.5 | 6850 | 4608 | 67.3 | **11.8** | 8.7 - 14.9 |

CI95%, 95% confidence interval; HCW, healthcare worker; PP, percentage point

**Supplementary Figure 1:** Boxplot (median, quartiles and ranges) of individual HH compliance both during baseline and intervention period

**Supplementary Figure 2.** Boxplot (median, quartiles and ranges) of hand hygiene increase (in percentage points) in the group of improving HCWs

**Supplementary Figure 3**: Variability within improving HCWs, measured as the range between the observation session with the lowest compliance and the session with the highest compliance for each HCW
